# eNAMPT induces alpha-cell mass expansion but impaired glucagon counter regulatory response

**DOI:** 10.1210/endocr/bqag061

**Published:** 2026-06-11

**Authors:** Sophie R Sayers, Vesela S Gesheva, Jithu J Varghese, Rebecca Beavil, Min Zhao, Yee Cheah, David Hopkins, Nicholas H F Fine, Annie Hasib, David J Hodson, Paul W Caton

**Affiliations:** Diabetes and Obesity Theme, School of Cardiovascular and Metabolic Medicine & Sciences, King's College London, London SE1 1UL, UK; Diabetes and Obesity Theme, School of Cardiovascular and Metabolic Medicine & Sciences, King's College London, London SE1 1UL, UK; Diabetes and Obesity Theme, School of Cardiovascular and Metabolic Medicine & Sciences, King's College London, London SE1 1UL, UK; Protein Production Facility, Randall Centre for Cell and Molecular Biophysics, King's College London, London SE1 1UL, UK; Diabetes and Obesity Theme, School of Cardiovascular and Metabolic Medicine & Sciences, King's College London, London SE1 1UL, UK; Diabetes and Obesity Theme, School of Cardiovascular and Metabolic Medicine & Sciences, King's College London, London SE1 1UL, UK; Diabetes and Obesity Theme, School of Cardiovascular and Metabolic Medicine & Sciences, King's College London, London SE1 1UL, UK; Institute of Metabolism and Systems Research (IMSR), and Centre of Membrane Proteins and Receptors (COMPARE), University of Birmingham, Birmingham B15 2TT, UK; Institute of Metabolism and Systems Research (IMSR), and Centre of Membrane Proteins and Receptors (COMPARE), University of Birmingham, Birmingham B15 2TT, UK; Department of Life Sciences, Manchester Metropolitan University, Manchester M15 6BH, UK; Oxford Center for Diabetes, Endocrinology and Metabolism (OCDEM), NIHR Oxford Biomedical Research Centre, Churchill Hospital, Radcliffe Department of Medicine, University of Oxford, Oxford OX3 7LE, UK; Diabetes and Obesity Theme, School of Cardiovascular and Metabolic Medicine & Sciences, King's College London, London SE1 1UL, UK

**Keywords:** NAMPT, beta-cells, alpha-cells, type 1 diabetes, type 2 diabetes, glucagon

## Abstract

**Context:**

Loss of functional beta-cell mass, coupled with alpha-cell dysfunction are key factors in pathophysiology of type 1 and type 2 diabetes. We have reported that extracellular nicotinamide phosphoribosyltransferase (eNAMPT) is elevated in type 2 diabetes and that elevated eNAMPT levels promote beta-cell dysfunction.

**Objective:**

To further investigate the effects of eNAMPT on beta-cell mass.

**Methods:**

Islets isolated from CD1 and Ins1^tm1.1(cre)Thor+/−^; mTmG^fl/−^ mice and human donors were exposed to eNAMPT (48-96 hours). CD1 mice were administered eNAMPT for 14 days. Alpha-, beta-, and delta-cell numbers were determined by glucagon, insulin, and somatostatin staining, respectively. Alpha-cell proliferation was assessed by bromodeoxyuridine (BrDU) uptake and Ki67 expression. Glucagon secretion was assessed via radioimmunoassay. Trans-differentiation was assessed by determining changes in presence of bi-hormonal cells in CD1/human islets and using Ins1^tm1.1(cre)Thor+/−^; mTmG^fl/−^ islets to determine changes in GLU+/GFP+ and GLU+/TdT+ cells.

**Results:**

eNAMPT treatment reduced beta-cell number and induced corresponding increases in alpha-cell number. Indicative of beta- to alpha-cell trans-differentiation eNAMPT induced increased presence of bi-hormonal INS+/GLU+ cells and PDX1+/GLU+ cells, and increased GLU+/GFP+ cells in Ins1^tm1.1(cre)Thor+/−^; mTmG^fl/−^ mouse islets. In addition, eNAMPT induced alpha-cell proliferation, indicated by increased BrDU uptake. Despite marked elevation in alpha-cell number, alpha-cell function was compromised following eNAMPT exposure, indicated by impaired glucagon counterregulatory response (CCR) to low glucose levels.

**Conclusion:**

This data supports a role for elevated eNAMPT levels in driving increased alpha-cell mass via a combination of beta- to alpha-cell trans-differentiation and alpha-cell proliferation. When combined with observed impaired CCR, these data have implications for both type 1 and type 2 diabetes pathophysiology.

Impaired pancreatic beta-cell function and mass is central to onset of both type 1 and type 2 diabetes. Type 2 diabetes (T2D) is characterized by progressive decline in functional beta-cell mass. This ultimately results in inadequate insulin secretion which cannot compensate for insulin resistance, with consequent onset of chronic hyperglycemia (1). Type 1 diabetes (T1D) is characterized by autoimmune mediated destruction of beta-cells leading to severe lack of insulin secretion and requirement of exogenous insulin therapy (2-6). Both conditions are also characterized by impaired alpha-cell function and glucagon secretion, which further contributes to disease pathophysiology (7-14). Developing a comprehensive understanding of the mechanisms underlying reduced beta-cell functional mass and impaired alpha-cell function is essential for identification of novel drug targets which can prevent or reverse decline in beta-cell mass in T2D and enhance residual beta-cell mass and function in T1D.

Recent studies have reported that de-differentiation and trans-differentiation of beta-cells are key pathophysiological mechanisms loss of beta-cell mass in T2D and to a lesser extent, T1D (15-21). De-differentiation involves beta-cells reverting to an embryonic and pre-endocrine progenitor cell phenotype, characterized by expression of eg, Ngn3, Oct4, Sox4, and Sox9. Trans-differentiation of beta-cells into alpha-cells is also reported. Additionally, both processes are characterized by reduced or loss of expression of key beta-cell identity genes, including PDX1, MAFA, and NKX6.1, leading to alterations in beta-cell specific transcription factor networks.

De-differentiation and trans-differentiation are thought to occur in part as defensive mechanisms to protect the beta-cell from inflammatory and glucolipotoxic attack and to protect against endoplasmic reticulum stress to avoid excess insulin production (22-24). Consequently, these processes are thought to be reversible and therefore attractive therapeutic targets (25). Understanding the mechanisms mediating de- and trans-differentiation are essential for realization of this translational potential.

Our previous studies have identified extracellular nicotinamide phosphoribosyltransferase (eNAMPT) as a key mediator of beta-cell dysfunction in T2D (26, 27). In nondiabetic individuals, eNAMPT circulates at ∼1 ng/mL and exists predominantly in dimeric form. Under this concentration-structure dynamic, eNAMPT enhances beta-cell function. However, in T2D serum eNAMPT circulates at >∼5 to 10 ng/mL and begins to adopt a monomeric form (27). Under this concentration-structure dynamic, eNAMPT mediates beta-cell failure via NAD-independent, pro-inflammatory mechanisms (Fig. 1A). Serum eNAMPT levels are also reportedly elevated in some T1D cohorts (28-31), indicating an additional potential pathophysiological role.

**Figure 1:**
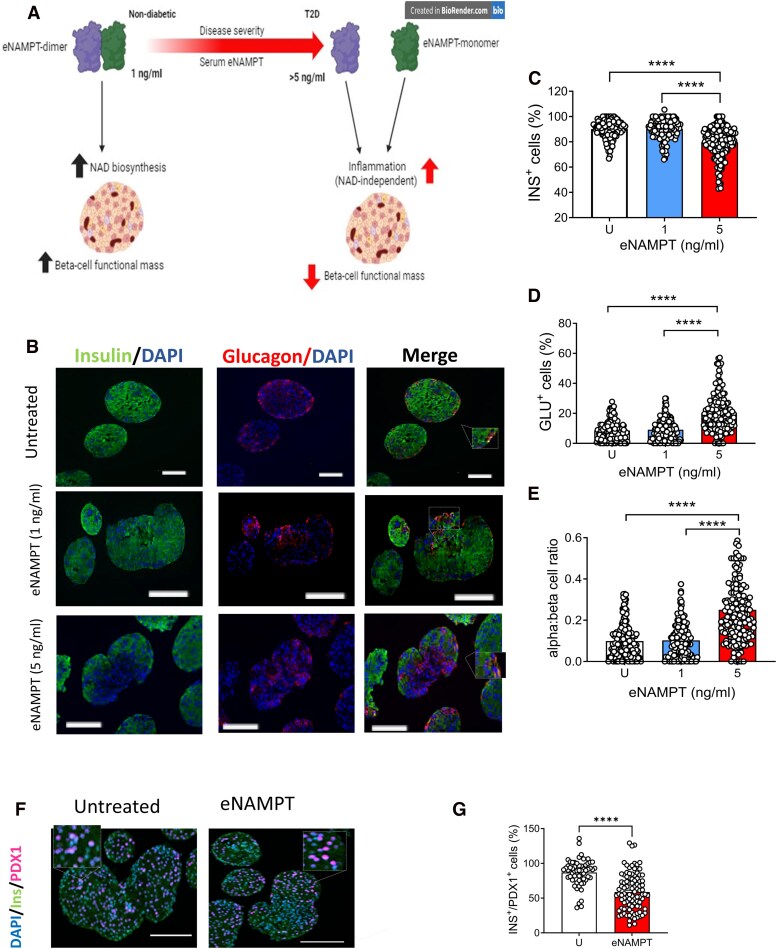
eNAMPT induces increased alpha- to beta-cell ratio in isolated mouse islets. (A) Diagrammatic representation of changes in eNAMPT structure-functional relationship at low and high serum concentration. Created in BioRender.com. (B-E) Islets were isolated from CD1 mice and treated with eNAMPT (1-5 ng/mL) for 96 hours. (B) Double immunofluorescence images of islets stained for insulin (green), glucagon (red), and DAPI (blue) (C) % DAPI+/INS+ stained cells; (D) % DAPI+/GLU+ stained cells; (E) alpha:beta cell ratio. N = 161 (untreated), 157 (1 ng/mL eNAMPT) and 189 (5 ng/mL eNAMPT) where n = 1 islet. (F-G) CD1 mouse islets were treated with eNAMPT (5 ng/mL) for 96 hours. (F) Double immunofluorescence images of islets stained for insulin (green), PDX1 (pink), DAPI (blue), and insulin (green), (G) % DAPI+/INS+/PDX1+ stained cells (N = 61 (untreated) and 97 (5 ng/mL eNAMPT) where n = 1 islet.). For all experiments, n = 3 mice/group were used for islet isolation. Data are expressed as means ± SEM; ****P < .0001, with bars indicating significance between specific experimental groups. (B-E) Significance was determined by 1-way ANOVA with Bonferroni post hoc test. (F-G) Significance was determined by nested *t*-test.

These data indicate that selectively targeting eNAMPT represents a potential therapeutic approach for improving beta-cell function in T2D and potentially T1D. However, drugs that solely target beta-cell function (eg, sulfonylureas) are reported to lose efficacy over time since they do not slow the chronic decline in beta-cell mass.

Consequently, elucidating the mechanistic impact of eNAMPT on beta-cell mass is important to determine the full pathophysiological importance and translational potential of eNAMPT as a diabetes drug target.

In this study, we set out to characterize the effects of eNAMPT on islet endocrine cell mass in rodent and human islets and in mice in vivo.

## Materials and methods

### Animals

For pancreatic islet isolation, 8-week-old male CD-1 mice (28-33 g; Envigo, Blackthorn, UK) or 8- to 12-week-old male and female Ins1^tm1.1(cre)Thor+/−^; mTmG^fl/−^ mice (32), with ad libitum access to standard mouse chow (Envigo) and water were used. For all animal experiments, mice were housed in 12 hours light/dark cycle, temperature-controlled conditions. Procedures were performed in accordance with UK Home Office regulations (Animal Scientific Procedures Act, 1986).

Studies with Ins1^tm1.1(cre)Thor+/−^; mTmG^fl/−^ mice were regulated by the Animals (Scientific Procedures) Act 1986 of the UK (Personal Project Licences P2ABC3A83 and PP1778740). Approval was granted by the University of Birmingham's and University of Oxford's Animal Welfare and Ethical Review Bodies (AWERB). All ethical guidelines were adhered to while carrying out this study. Mice were socially housed in specific pathogen-free conditions at Biomedical Services Unit, University of Birmingham, under a 12-hour light-dark cycle with ad libitum access to food and water. Relative humidity was 55% ± 10% and temperature 21 ± 2 °C.

For experimental elevation of eNAMPT, 8-week-old male CD1 mice were intraperitoneally injected daily with recombinant eNAMPT or the equivalent volume of NaCl 154 mmol/L (Sigma) for 14 days (*n* = 6/group) in order to elevate plasma eNAMPT to 5 to 10 ng/mL, to replicate conditions observed in diabetes. After 14 days, mice were sacrificed and whole pancreas was collected. Plasma glucagon was measured by enzyme-linked immunosorbent assay (Crystal Chem, IL, USA; RRID: AB_2811007)

Mice were maintained on a 12 hours light/12 hours dark cycle. Animal experiments were conducted in accordance with UK Home Office Animals (Scientific Procedures) Act 1986, with local ethical committee approval. Experimenters were not blinded to group assignment or outcome assessment. Specific randomization of animals into groups was not carried out. No data, samples, or animals were excluded from the study.

### Pancreatic islet isolation and treatment

Mouse islets were isolated between 9:00 and 10:00 hours, as previously described (27). Briefly, pancreases were inflated with 1 mg/mL collagenase solution (Sigma-Aldrich, Poole, UK) followed by density gradient separation (Histopaque-1077; Sigma-Aldrich). Human islets were isolated from heart-beating nondiabetic donors (Table S1; 10.6084/m9.figshare.30607520 (33)) at the King's College Hospital Human Islet Isolation Unit (London, UK) and made available for research via the KCL Human Islet Research Tissue Bank (20/SW/0074). All isolated islets were incubated overnight (37 °C, 5% CO_2_) prior to treatments. RPMI media was used for all incubations. The islets were matched prior to treatments. Mouse and human islets were treated with wild-type eNAMPT (1-5 ng/mL), generated as described in (27) for 48 to 96 hours. eNAMPT protein was prepared in saline and further dissolved in RPMI media to generate final concentrations. Controls were islets treated with RPMI media only. For bromodeoxyuridine (BrDU) uptake experiments, isolated mouse islets were incubated with 1 mg/mL BrDU in RPMI (supplemented with 2% FBS, 2mM L-glutamine and 100 U/mL penicillin/0.1 mg/mL streptomycin) for 168 hours. The eNAMPT treatments were added for the final 96-hours of the 168-hours incubation. Islets were then fixed and BrDU uptake was assessed using immunohistochemistry using a mouse monoclonal anti-BrDU antibody; RRID:AB_476793.

### Islet glucagon secretion

CD1 mouse islets were pre-incubated in a physiological salt solution (Gey and Gey Buffer; 111mM NaCl, 5mM KCL, 27mM NaHCO_3_, 1mM MgCl_2_, 0.22mM KH_2_PO_4_, 0.28mM MgSO_4_) containing 2mM glucose. Groups of 3 to 5 size-matched islets were then further incubated at 37 °C for 1 hour in salt solution and 10 mmol/L glucose. Following this, islets were incubated with either 3, 10, or 20 mmol/L glucose for a further 1 hour. Secreted glucagon was measured using a radioimmunoassay (Merck).

### Islet immunofluorescence histochemistry

Islets were incubated with indicated concentrations of eNAMPT for 48 to 96 hours. CD1 mice were administered eNAMPT daily for 14 days (intraperitoneal). Following treatments, isolated islets/pancreas were fixed in 4% (vol/vol) buffered formalin and underwent a series of ethanol and xylene/histoclear washes before being embedded in paraffin wax. Sections were then cut at 5 μm on superfrost slides. Slides were submerged in xylene or histoclear followed by washing in decreasing concentrations of ethanol to remove paraffin wax. Permeabilized islets were blotted with primary antibodies (all 1:100; see Table 1) and were visualized by subsequent incubation with Alexa Fluor antibodies (all 1:200; see Table 1). DAPI was used to stain nuclei (1:1000, Sigma-Aldrich). Samples were mounted on glass slides using Fluoromount Aqueous Mounting Medium (Dako). Images were captured on a Nikon Ti2 wide-field microscope and fluorescent quantification was achieved using Image J.

**Table 1:** Details of primary and secondary antibodies used in the study

Primary antibodies				
Target	Host	Supplier	Catalog number	RRID
INSULIN	Guinea pig	Dako	AO564	AB_10013624
GLUCAGON	Rabbit	Abcam	ab92517	AB_10561971
PDX1	rabbit	Abcam	ab219207	AB_2891187
ALDHA1A3	Rabbit	Novus	NBP3-12280	AB_3586410
LY6A	rat	Abcam	ab51317	AB_1640946
KI67	rabbit	Abcam	ab15580	AB_805388
SOMATOSTATIN	Rat	Abcam	ab30788	AB_778010
NGN3	RABBIT	MILLIPORE	AB10535	AB_10618738
ChGA	RABBIT	Abcam	EPR22537-248	AB_2910555
BrDU	MOUSE	Sigma	B2531	AB_476793

Secondary antibodies			
Target	Supplier	Catalog number	
AlexaFluor 488-labeled donkey anti-rat	Jackson ImmunoResearch	712-545-150	AB_2340683
AlexaFluor 594-labeled donkey anti-guinea pig	Jackson ImmunoResearch	706-585-148	AB_2340474
AlexaFluor 488-labeled donkey anti-rabbit	Jackson ImmunoResearch	711-545-152	AB_2313584

### Quantitative reverse-transcriptase polymerase chain reaction

Total human or mouse islet RNA was extracted using Trizol reagent (Invitrogen, Paisley, UK). Reverse transcription was performed using the High-Capacity cDNA reverse transcription kit (Applied Biosystems, Warrington, UK). Real-time quantitative polymerase chain reaction (qPCR) was carried out with a LightCycler480, using Sybr Green PCR master mix (Qiagen, Hilden, Germany). Gene expression was measured by ΔΔCt methodology, normalized against GAPDH (Quantitech, UK).

### Statistical analysis

Data are expressed as mean ± SEM. Significance was tested using either one-way ANOVA with Tukey's post hoc test, unpaired *t*-test, or nested *t*-test. Outlier testing was conducted using ROUT methodology with Q (False Discovery Rate) set at 1%. All statistics testing was conducted using GraphPad PRISM 10 software (San Diego, CA, USA).

## Results

### eNAMPT induces increased alpha- to beta-cell ratio in isolated mouse islets

To determine the effects of eNAMPT on islet cell mass, isolated CD1 mouse islets were treated with 1 or 5 ng/mL eNAMPT and stained using immunohistochemistry for glucagon (GLU) or insulin (INS) to determine alpha- and beta-cell mass, respectively. Consistent with previous studies of eNAMPT on beta-cell health, no changes in INS^+^ and GLU^+^ cells (Fig. 1B-1D) were observed following treatment with “physiological” concentrations of eNAMPT (1 ng/mL) similar to those observed in nondiabetic individuals. In contrast, pathophysiological eNAMPT concentrations (5 ng/mL) induced a marked reduction in numbers of INS^+^ cells together with a corresponding increase in GLU^+^ cells, indicating reduced beta-cell mass and increased alpha-cell mass in response to eNAMPT exposure at concentrations typically observed in diabetic serum (Fig. 1B-1D). Consistent with this, we also observed increased alpha- to beta-cell ratio in 5 ng/mL eNAMPT-treated islets (Fig. 1E). Pathophysiological levels of eNAMPT also induced changes in the beta-cell enriched marker PDX1, with eNAMPT exposure leading to reductions in numbers of PDX1^+^/INS^+^ cells (Fig. 1F and 1G). Together, these data demonstrate that exposure of islets to “diabetic” concentrations of eNAMPT leads to reductions in beta-cell mass, and corresponding increases in alpha-cell number.

### eNAMPT treatment increases alpha-cell number in human islets and in vivo

**Figure 2:**
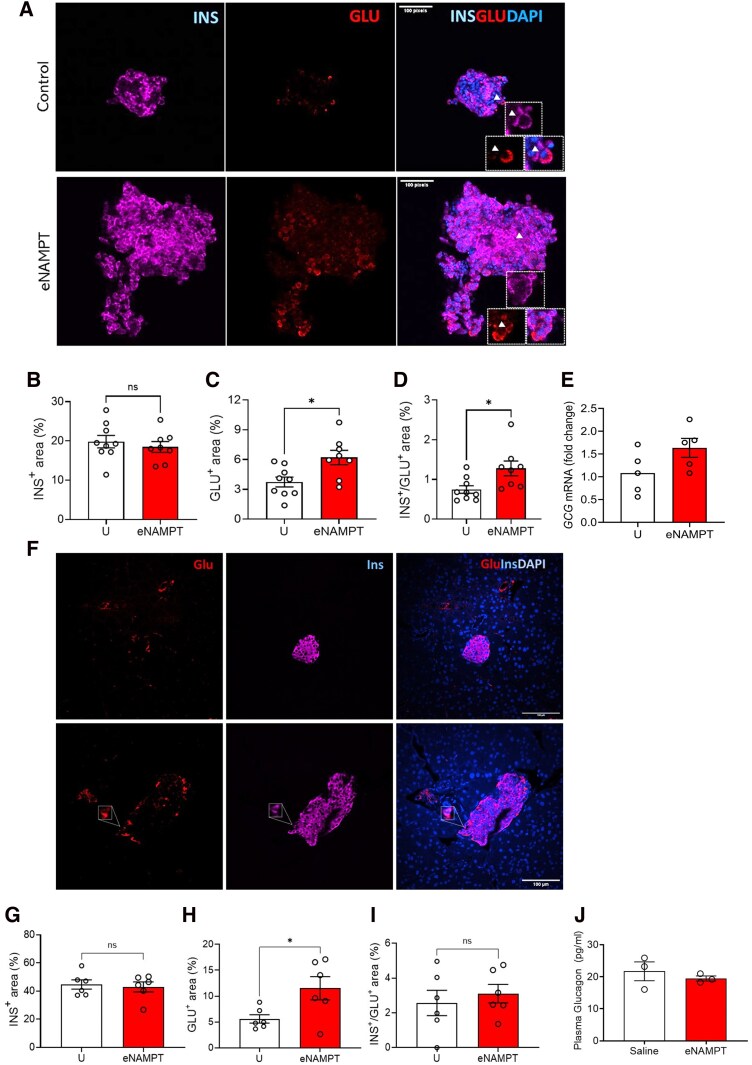
Effects of elevated eNAMPT in isolated human islets and in vivo in CD1 mice. (A-D) Isolated human islets were treated with eNAMPT (5 ng/mL; 48 hour), fixed and stained for insulin (purple), glucagon (red), and DAPI (blue). (B) % DAPI+/INS+ stained area; (C) % DAPI+/GLU+ stained area (D) INS+/GLU+ area. Quantification for each whole islet/image was carried out manually using ImageJ, n = 7 to 9 whole islets per group (approx. 10-20 z-stack slices per islet). (E) Glucagon (GCG) mRNA was determined by qPCR. For A-E, islets were obtained from 2 different donors (F-J) 8-week-old male CD1 mice were administered eNAMPT or saline control (IP) daily for 14 days, to elevated plasma eNAMPT levels to 5 to 10 ng/mL. (F) After 14 days, whole pancreas was extracted, fixed, and stained for insulin (purple), glucagon (red), MAFB (green), and DAPI (blue). (G) %DAPI+/INS+ stained area; (H) % DAPI+/GLU+ stained area (I) INS+/GLU+ area. Representative 40× magnification images are shown consist of 10 z-stack slices merged at max intensity. The scale bar represents 100 mm. (J) Plasma glucagon levels were measured by enzyme-linked immunosorbent assay. Data are expressed as means ± SEM, (n = 3-6, where n = 1 mouse). *P < .05; with bars indicating significance between specific experimental groups. Significance was determined by nested *t*-test.

To corroborate findings in mouse islets described above, we examined the effects of elevated eNAMPT levels in isolated human islets and in nondiabetic CD1 mice in vivo. Isolated human islets were treated with 5 ng/mL eNAMPT and stained using immunohistochemistry for glucagon (GLU) or insulin (INS) to determine alpha- and beta-cell mass, respectively. Although numbers of INS+ cells were overall unchanged, eNAMPT treatment induced a significant increase in GLU^+^ cells (*P* < .05), indicating increased alpha-cell mass in response to eNAMPT exposure (Fig. 2A-2C). Consistent with increased occurrence of beta- to alpha-cell trans-differentiation, we also observed increased numbers of bi-hormonal INS+/GLU+ cells in response to eNAMPT treatment (Fig. 2D; *P* < .05). GCG (glucagon) mRNA levels were unchanged (Fig. 2E). To assess effects of increased eNAMPT in vivo, CD1 mice were administered eNAMPT (or saline control) via daily intraperitoneal administration for 14 days, in order to elevate plasma eNAMPT levels to 5 to 10 ng/mL, as observed in diabetic models. Similar to effects observed in human islets, eNAMPT administration to CD1 mice induced increases in GLU^+^ cells (*P* < .05) without concomitant changes in INS^+^ cell number (Fig. 2F-2H). However, in vivo, we did not observe changes in bi-hormonal cell number (Fig. 2I) suggesting that trans-differentiation is not occurring, at least in response to 14 days eNAMPT elevation. Increases in alpha-cell number were not accompanied by elevated plasma glucagon levels, potentially suggesting impaired alpha-cell function in vivo in response to eNAMPT elevation (Fig. 2J).

### eNAMPT-mediated alpha-cell expansion occurs via beta- to alpha-cell trans-differentiation and enhanced alpha-cell proliferation

**Figure 3:**
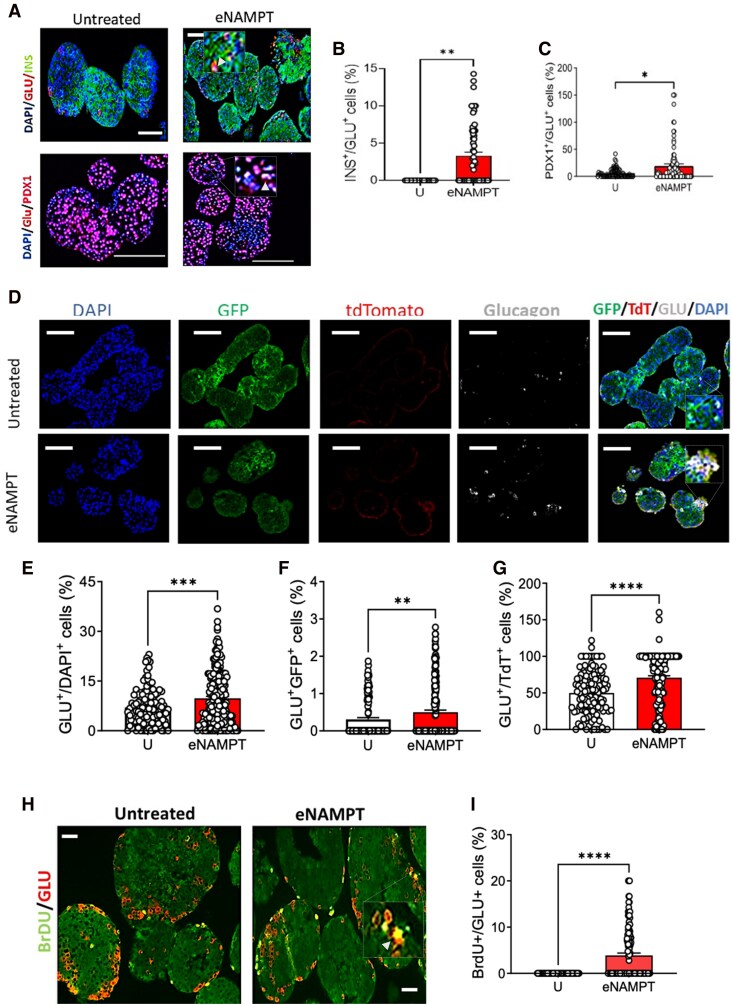
eNAMPT increases alpha-cell mass via beta- to alpha-cell trans-differentiation and alpha-cell proliferation. (A-C) Islets were isolated from CD1 mice and treated with eNAMPT (5 ng/mL) for 96 hours. (A) Double immunofluorescence images of islets stained for insulin (green), glucagon (red), DAPI (blue); and double immunofluorescence images of islets stained for glucagon (red), PDX1 (pink), DAPI (blue). (B) % DAPI+/INS+/GLU+ stained cells (N = 21 [untreated] and 67 [5 ng/mL eNAMPT] where n = 1 islet). (C) % DAPI+/GLU+/PDX1+ stained cells (N = 93 [untreated] and 25 [5 ng/mL eNAMPT] where n = 1 islet). (D-G) Islets were isolated from mTmG mice and treated with eNAMPT (5 ng/mL) for 96 hours. (D) Immunofluorescence images of islets stained for DAPI (blue), glucagon (gray), GFP (green), and TdTomato (red); (E) % GLU+/DAPI+ stained cells; (F) % GLU+/GFP+ stained cells; (G) % GLU+/TdTomato+ stained cells (for F-H N = 131 [untreated] and 208 [5 ng/mL eNAMPT] where n = 1 islet). (H-I) Islets were isolated from CD1 mice and treated with BrDU for 168 hours, with eNAMPT (5 ng/mL) added for the final 96 hours. (H) Immunofluorescence images of islets stained for BrDU (light green), and glucagon (red), (I) % GLU+/BrDU+ stained cells. For all experiments, n = 3 mice/group were used for islet isolation. Data are expressed as means ± SEM, *P < .05; **P < .01; ***P < .001; ****P < .0001, with bars indicating significance between specific experimental groups. Significance was determined by unpaired *t*-test.

To understand the mechanisms that mediate changes in alpha- to beta-cell ratio, we began by assessing the possibility of direct beta- to alpha-cell trans-differentiation. Consistent with this hypothesis, we observed a 3-fold increase in numbers of bi-hormonal INS^+^/GLU^+^ cells in CD1 islets treated with eNAMPT (Fig. 3A and 3B; *P* < .01). We also noted the appearance of GLU+ cells in the center of eNAMPT-treated islets. In mouse islets, alpha-cells are primarily located around the islet periphery. The presence of GLU+ cells away from the periphery could further support the notion that increased GLU+ cells occur due to conversion from beta-cells (Fig. 3A). Moreover, eNAMPT exposure led to an apparent increase in misexpression of the beta-cell identity marker and transcription factor PDX1, as indicated by a 3-fold increase in numbers of CD1 islet cells which were GLU^+^/PDX1^+^ (Fig. 3A and 3C; *P* < .05). Together these findings show formation of bi-hormonal cells expressing both insulin and glucagon, together with misexpression of PDX1, both of which point toward potential beta- to alpha-cell trans-differentiation. To further examine whether eNAMPT induces beta- to alpha-cell trans-differentiation, we utilized Ins1^tm1.1(cre)Thor+/−^; mTmG^fl/−^ mice to conduct lineage-tracing experiments and directly map beta-cell fate. Membrane-TdTomato/Membrane-GFP mice (mT/mG) express TdTomato in all cells until Cre-mediated recombination, when membrane GFP becomes expressed. When crossed with Ins1Cre mice, which express Cre solely in the insulin gene, Cre-mediated recombination occurs specifically in beta-cells. Thus, in Ins1^tm1.1(cre)Thor+/−^; mTmG^fl/−^ beta-cells are marked with GFP and all other islet cells are marked with TdTomato fluorescent protein (32). This allows beta-cell fate to be mapped by GFP staining even when insulin expression is lost. When islets were isolated from mTmG mice and treated with eNAMPT for 96 hours, we observed an overall increase in GLU^+^ cells (3-fold; *P* < .001; Fig. 3D and 3E). Within the GLU^+^ cell population, we observed a significant increase in GLU^+^/GFP^+^ cells (*P* < .01; Fig. 3D and 3F). This represents a population of cells that were beta-cells prior to trans-differentiation into alpha-cells. In addition, we also observed increases in GLU^+^/TdT^+^ cells (*P* < .0001; Fig. 3D and 3G), representing a population of alpha-cells that were not formed via trans-differentiation. To investigate alternative non-trans-differentiation mechanisms which may be responsible for the increases in GLU^+^/TdT^+^ cells, CD1 islets were co-treated with eNAMPT and BrDU to examine alpha-cell proliferation. We observed a marked increase in alpha-cell proliferation, as shown by a 4-fold increase in BrDU^+^/GLU^+^ cells, representing BrDU uptake into alpha-cells (Fig. 3H and 3I; *P* < .0001). Separately, we also observed a nonsignificant trend toward increased numbers of Ki67^+^/GLU^+^ cells following eNAMPT treatment (Fig. S1A-S1C; 10.6084/m9.figshare.30607520 (33)), further suggesting a role for increased proliferation in inducing alpha-cell mass expansion. Delta-cell numbers were unchanged in response to eNAMPT treatment, as demonstrated by unchanged numbers of SST^+^ cells, and unchanged delta- to beta-cell ratio (Fig. S2A-S2C; 10.6084/m9.figshare.30607520 (33)).

Together, this data demonstrates that pathophysiological eNAMPT exposure induces a marked expansion of the islet alpha-cell population, mediated by both beta- to alpha-cell trans-differentiation and alpha-cell proliferation.

### Effects of eNAMPT on beta-cell de-differentiation

**Figure 4:**
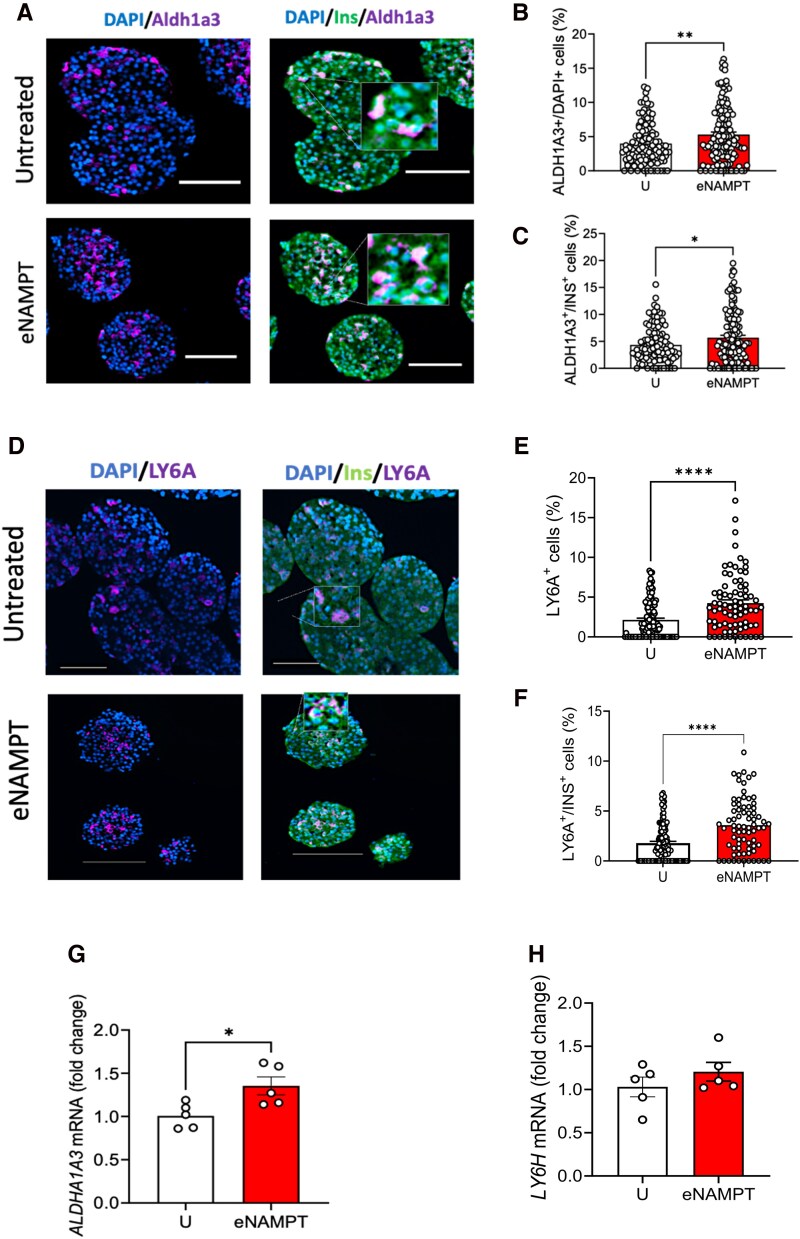
eNAMPT increases beta-cell expression of ALDH1A3 and Ly6G. (A-C) Isolated human islets were treated with eNAMPT (5 ng/mL) for 48 hours. (A) Double immunofluorescence images of islets stained for ALDH1A3 (purple), insulin (green), and DAPI (blue); (B) % DAPI+/ALDH1A3+ stained cells; (C) % DAPI+/ALDH1A3+/INS+ stained cells (for B and C; N = 135 [untreated] and 114 [eNAMPT] where n = 1 islet). (D-F) Islets were isolated from CD1 mice and treated with eNAMPT (5 ng/mL) for 96 hours. (D) Double immunofluorescence images of islets stained for LY6A (purple), insulin (green), and DAPI (blue); (E) % DAPI+/LY6a+ stained cells; (F) % DAPI+/LY6A+/INS+ stained cells (for B and C; N = 78 [untreated] and 129 [eNAMPT] where n = 1 islet). (G-H) Isolated human islets were treated with 5 ng/mL eNAMPT and (G) ALDH1A3 and (H) LY6A mRNA levels were measured by qPCR (n = 5/group). For all mouse experiments, n = 3 mice/group were used for islet isolation. For human islets, islets were obtained from 2 different donors. Data are expressed as means ± SEM, *P < .05; **P < .01, with bars indicating significance between specific experimental groups. (A-C, E-F) Significance was determined by unpaired *t*-test. (G, H) Significance was determined by nested *t*-test.

Beta-cell de-differentiation is thought to be a key mechanism leading to reduced beta-cell mass in T2D and T1D. Several studies have reported de-differentiation as being characterized by loss of beta-cell identity markers such as PDX1, as well as re-expression of endocrine and pre-endocrine progenitor markers (15-18, 21). Some studies report beta-cell de-differentiation followed by re-differentiation into alpha- or delta-cells (15). We used islets isolated from mTmG mice to investigate whether eNAMPT-mediated loss of beta-cell mass was linked to beta-cell de-differentiation, by staining recognized markers of endocrine progenitor cells (NGN3) and committed endocrine cells (ChgA). However, these experiments did not provide evidence of eNAMPT-mediated beta-cell de-differentiation, as indicated by the lack change in either NGN3^+^/GFP^+^ (Fig. S3A-S3C; 10.6084/m9.figshare.30607520 (33)) nor ChgA^+^/GFP^+^ cells (Fig. S4D and S4E; 10.6084/m9.figshare.30607520 (33)), between untreated and eNAMPT-treated islets. However, eNAMPT treatment did lead to increases in ChgA^+^/TdT^+^ cells (Fig. S3D-S3F; *P* < .0001; 10.6084/m9.figshare.30607520 (33)), indicating potential increases in pre-endocrine progenitor cells and committed endocrine cells derived via mechanisms other than beta-cell de-differentiation. These findings are consistent with recent studies in models of T1D and T2D which failed to show beta-cell re-expression of classical de-differentiation markers and instead reported re-expression of LY6A and ALDH1A3 in T1D and T2D models, respectively (20). Therefore, we investigated the impact of eNAMPT on ALDH1A3 and LY6A. Treatment with eNAMPT led to increased numbers of total ALDH1A3^+^ cells and ALDH1A3^+^/INS^+^ in isolated human islets (Fig. 4A-4C). The eNAMPT induced increases in total numbers of Ly6A^+^ and Ly6A^+^/INS^+^ cells in CD1 mouse islets (Fig. 4D-4F). The eNAMPT also increased ALDH1A3 mRNA levels in isolated human islets (Fig. 4G) but had no effect on Ly6A mRNA in human islets exposed to eNAMPT (Fig. 4H). Together this data indicates that eNAMPT induces increased expression ALDAH1A3 and LY6A in beta-cells, suggesting that eNAMPT may play a role in pathophysiology of T1D and T2D by induction of beta-cell de-differentiation leading to decline in functional beta-cell mass.

### Alpha-cells are functionally compromised following eNAMPT exposure

**Figure 5:**
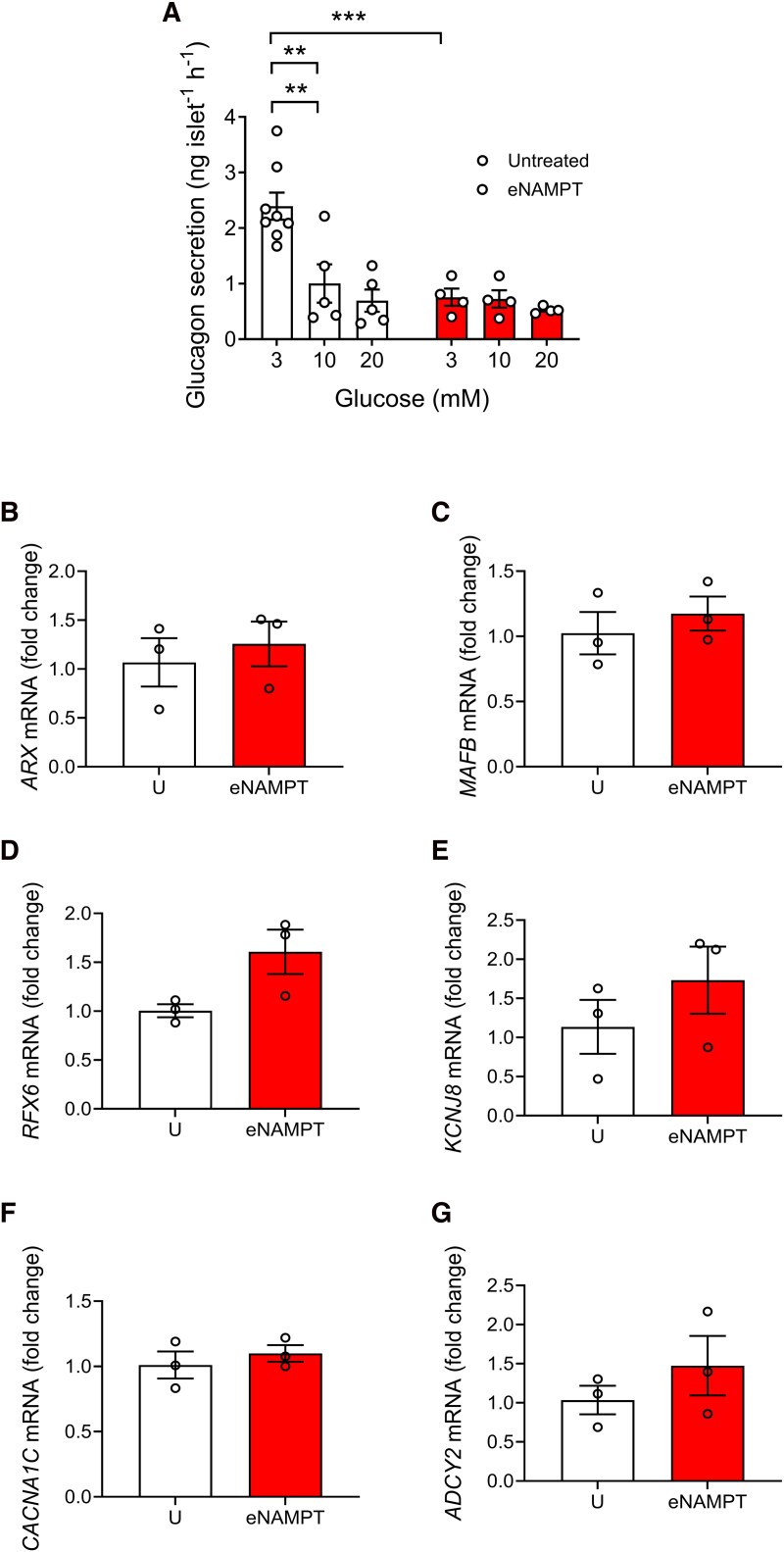
Alpha-cell function is impaired following eNAMPT exposure. Islets were isolated from CD1 mice and treated with eNAMPT (5 ng/mL) for 96 hours. (A) Static glucagon secretion in response to 3mM, 10mM and 20mM glucose was assessed in isolated mouse islets (n = 4-8/group). (B-G) Gene expression of (B) ARX, (C) MAFB, (D) RFX6, (E) KCNJ8, (F) CACNA1C, and (G) ADCY2 were measured by qPCR. For all experiments, n = 3 mice/group were used for islet isolation. Data are expressed as means ± SEM, **P < .01; ***P < .001; ****P < .0001, with bars indicating significance between specific experimental groups. (A) Significance was determined by two-way ANOVA with Tukey's post-test (B-G) Significance was determined by unpaired *t*-test.

We next examined the functionality of the expanded alpha-cell population, reasoning that abnormally elevated glucagon secretion may mediate some of the contribution of eNAMPT to diabetes pathophysiology. However, despite a 2-fold expansion in alpha-cell mass in response to eNAMPT exposure (Fig. 1H), this was not reflected in increased alpha-cell functionality. Glucagon secretion was significantly decreased following eNAMPT treatment, indicative of impaired alpha-cell function. Specifically, CD1 islet glucagon secretion in response to 3 mmol/L glucose was approximately 3-fold less in eNAMPT-treated islets compared to untreated islets. However, there was no difference in glucagon secretion following incubation with either 10 or 20 mmol/L glucose between the eNAMPT-treated and untreated groups (Fig. 5A). Thus, normal glucagon counterregulatory response (CRR) to low glucose was significantly impaired following eNAMPT treatment. Similar defective glucagon secretion has been reported in islets obtained from T1D donors (34). We next examined the impact of eNAMPT on mRNA levels of the alpha-cell enriched transcription factors ARX and MAFB. Despite elevated alpha-cell number, ARX and MAFB mRNA levels were unchanged in response to eNAMPT exposure (Fig. 5B and 5C; (33)). Downstream targets of ARX and MAFB include genes encoding for key proteins involved in glucagon secretion (34). Interestingly, eNAMPT exposure had no effect on mRNA levels of genes involved in alpha-cell identity (RFX6), cAMP signaling (*ADCY2*), potassium channel function (*KCNJ8*) and calcium channel function (*CACNA1C*) (Fig. 5D-5G; 10.6084/m9.figshare.30607520 (33)). Thus, eNAMPT-mediated increases in alpha-cell number were not accompanied by corresponding increases in key alpha-cell transcription factors nor in genes encoding proteins involved in glucagon secretion, potentially explaining the impaired glucagon secretion observed in this model.

## Discussion

This study demonstrates that pathophysiological levels of eNAMPT, similar to those observed in diabetic serum, induce reductions in beta-cell number with corresponding expansion of alpha-cell and delta-cell mass in mouse islets. eNAMPT-mediated reductions in beta-cell mass were driven by a combination of beta-cell de-differentiation and trans-differentiation. The latter mechanism, together with a marked increase in alpha-cell proliferation, was responsible for associated increases in alpha-cell number. Evidence of alpha-cell expansion and beta- to alpha-cell trans-differentiation in response to eNAMPT was also observed in isolated human islets.

Serum eNAMPT levels are commonly elevated in T2D (27, 29, 35), with some studies also reporting eNAMPT elevation in T1D serum (28-31). Our previous studies have reported that pathophysiological levels of eNAMPT can induce beta-cell dysfunction, although the precise mechanisms remained unclear (27). The effects of eNAMPT on beta-cell failure in T1D remain unexplored.

The current study suggests that eNAMPT can induce loss of beta-cell mass via beta-cell de-differentiation and trans-differentiation. eNAMPT exposure led to a marked increase in ALDA1A3 in human islets and LY6A in mouse islets. Recent studies have described distinct de-differentiation pathways, with ALDA1A3 and Ly6A expression lost during beta-cell differentiation and re-expressed in mature beta-cells in models of T2D and T1D, respectively (20). Thus, the data presented here suggests that eNAMPT exposure can potentially induce beta-cell de-differentiation consistent with both T1D and T2D specific islet phenotypes, indicating a pathophysiological role in both conditions. The lack of increase in beta-cell expression of classical de-differentiation markers NGN3 and ChgA observed following eNAMPT treatment is also consistent with the findings of the study described above, which did not observe alterations in these markers in T1D nor T2D models (20).

Together with loss of beta-cell mass, eNAMPT induced a corresponding increase in alpha-cell number. Alpha-cell dysfunction and increased alpha-cell mass are also characteristic of T1D and T2D (7-14, 36). We report here that eNAMPT induces a marked increase in mouse alpha-cell number, driven by a combination of beta- to alpha-cell trans-differentiation and alpha-cell proliferation. However, alpha-cell expansion following eNAMPT exposure was accompanied by impaired alpha-cell function, whereby glucagon secretion was not increased in response to incubation with low glucose levels. These findings were partially confirmed in isolated human islets, where eNAMPT exposure induced increased alpha-cell number and increased INS+/GLU+ bi-hormonal cells, and in CD1 mice in vivo, where elevated plasma eNAMPT corresponded with increased alpha-cell number, but no change in plasma glucagon levels. While impaired glucagon secretion can occur in severe T2D, this phenotype is more consistent with that commonly reported in T1D, whereby the glucagon counterregulatory response (CRR) to low glucose levels is commonly impaired, despite increased alpha-cell mass, resulting in increased risk of hypoglycemia (7-9, 34). Compromised CRR was associated with unchanged levels of in the key regulatory transcription MAFB and ARX, despite increases in alpha-cell number (34). These transcription factors and others, including RFX6, regulate transcription of genes involved in alpha-cell intra-cellular signaling and glucagon exocytosis, and a lack of corresponding increase in MAFB and ARX levels in our model provide a potential mechanistic underpinning for impaired glucagon secretion (34). These findings point to a potential role for elevated eNAMPT levels as a driver of alpha-cell dysfunction, leading to impaired CRR to hypoglycemia in T1D. Hypoglycemia is a common and often life-threatening complication in individuals with T1D, and to a lesser extent T2D, who require exogenous insulin therapy. Multiple hypoglycemic episodes are also linked to long-term neurocognitive impairments, and increased psychological burden (eg, anxiety, depression) (8, 37). Moreover, hypoglycemia is estimated to be responsible for 6% to 10% of T1D-associated mortality (8). Despite this, no therapies capable of preventing hypoglycemia are currently approved for use. Our data indicates that therapies which block the action of eNAMPT could be attractive therapeutic approaches to prevent hypoglycemia in individuals with T1D.

Several different mechanisms have been suggested to mediate impaired alpha-cell CRR, including intrinsic alpha-cell secretory defects, alpha-cell insensitivity to glucose, impaired beta- to alpha-cell paracrine signaling (mediated by eg, insulin, GABA) occurring secondary to beta-cell destruction, and excess delta-cell somatostatin secretion (10, 11, 34, 38, 39). eNAMPT has the potential to influence several of these mechanisms. Lack of concomitant increase in ARX/MAFB despite alpha-cell expansion are consistent with intrinsic alpha-cell defects (34), driven by a direct effect of eNAMPT on alpha-cells. However, the current data also show that eNAMPT can also mediate loss of beta-cell mass, suggesting impaired beta- to alpha-cell communication which may also mediate alpha-cell dysfunction. The data presented here has focused on chronic exposure of islets to elevated levels of eNAMPT, as observed in disease. Further studies will be required to determine how eNAMPT potentially impacts glucagon secretion during circadian eNAMPT fluctuations and in response to acute increases in eNAMPT.

The mechanisms by which eNAMPT mediates de-differentiation, trans-differentiation, and alpha-cell proliferation are likely linked to onset of inflammation and potentially reductions in NAD levels. Our previous studies have demonstrated that the consequences of the structure-functional switch from dimeric to monomeric eNAMPT are a reduction in eNAMPT-dimer-mediated islet NAD synthesis and an increase in eNAMPT-monomer-mediated islet inflammation (27). Islet inflammation, and associated oxidative and ER stress, has been reported to mediate beta-cell de-differentiation (40), with altered differentiation status potentially occurring as a mechanism to protect beta-cells against stress. Hence, changes in differentiation status reported here may occur secondary to eNAMPT-mediated inflammation and apoptosis. The underlying signaling mechanisms that mediate monomeric eNAMPT function in pathophysiological situations have yet to be elucidated. Interactions between eNAMPT and cell surface receptors likely play a role, however these remain incompletely characterized. Several studies have reported that eNAMPT acts via binding to TLR4 (41), although not all studies have replicated this. Our own studies in isolated islets failed to find any effects of TLR4 antagonism on eNAMPT-mediated beta-cell failure (42). We observed that eNAMPT-mediated beta-cell failure was linked to STAT3 activation (27), which potentially implicates numerous receptor signaling cascades in eNAMPT action, including several cytokine and growth factor receptors; however, these possibilities have yet to be examined in detail. Other studies have identified CCR5, BDKRB1 and BDKRB1, CD44, GPRC5B, HCRTR2, LRP8, PEAR1, and CBL, as possible eNAMPT receptors, suggesting that eNAMPT may be able to target multiple cell surface receptors (43).

Conversely, several enzymes and transcription factors that underpin maintenance of islet endocrine cell identity are regulated by NAD. For example, NAD has been shown to regulate levels and activity of FOXO1 (44) and DMNT1 (45, 46), which are reported to play a key role in maintenance of beta-cell identity and differentiation status. Moreover, NAD boosting strategies, including our own work examining the effects of eNAMPT dimer on islets (27, 47), have been reported to enhance beta-cell mass and increase levels of beta-cell identity markers such as PDX1. Consequently, reductions in cellular NAD levels mediated by a switch from dimeric to monomeric eNAMPT would be predicted to result in lower islet NAD levels and reductions in PDX1 levels and loss of beta-cell identity.

There are several limitations to the current study. These include lack of information on sex-specific differences that could impact the findings; small sample sizes for some experiments; the fact that mouse results were only corroborated with human islets for some experiments, and that across the different model systems phenotypes did always match. All of these limitations potentially limit the wider significance of the data, and further studies are required to fully understand the phenotypes observed.

## Conclusion

Taken together, these data indicate that elevated eNAMPT can mediate loss of beta-cell mass together with increased numbers of poorly functional alpha-cells. These effects are driven in part through beta-cell de-differentiation, beta-cell to alpha-cell trans-differentiation, and alpha-cell proliferation. These data provide novel information detailing the mechanistic effects of eNAMPT in diabetes pathophysiology, suggesting that eNAMPT exposure can induce effects on pancreatic islets that are consistent with both a T1D and T2D phenotype. Future studies will determine the potential for specific interactions between eNAMPT and other T1D- or T2D- disease specific factors which may mediate observed effects. Overall, the data indicate a potential therapeutic role for selective inhibitors of eNAMPT for both preventing or slowing decline in functional beta-cell mass and for restoring alpha-cell CRR to prevent hypoglycemia following insulin therapy.

## Data Availability

Original data generated and analyzed during this study are included in this published article or in the data repositories listed in References.

## Abbreviations

CRR: counterregulatory response
eNAMPT: extracellular nicotinamide phosphoribosyltransferase
GLU: glucagon
INS: insulin
qPCR: quantitative polymerase chain reaction
T1D: type 1 diabetes
T2D: type 2 diabetes
